# Deciphering Alkaloid Bitter Compounds and Relevant Transcription Factors in Papaya

**DOI:** 10.3390/ijms27083438

**Published:** 2026-04-11

**Authors:** Jiayi Kong, Yutong Zheng, Jianling Pan, Zhihui Yang, Yuru Tang, Mengjun Xiao, Ray Ming

**Affiliations:** Center for Genomics and Biotechnology, Fujian Provincial Key Laboratory of Haixia Applied Plant Systems Biology, Key Laboratory of Genetics, Breeding and Multiple Utilization of Corps, Ministry of Education, Fujian Agriculture and Forestry University, Fuzhou 350002, China18276811148@163.com (J.P.);

**Keywords:** papaya, bitter alkaloids, fibrous strands, regulatory genes

## Abstract

Papaya (*Carica papaya* L.) is a widely cultivated tropical and subtropical fruit crop valued for its rich nutritional content, diverse food industry applications, and the medicinal use of papain. However, bitterness in papaya fruit, particularly in fibrous strands, negatively affects fruit quality and consumer acceptance; therefore, the development of papaya cultivars with stable and desirable quality is of great importance. To identify the bitter compounds in papaya fruit fibrous strands and elucidate the molecular mechanisms underlying their biosynthesis, we performed transcriptomic and metabolomic analyses of fibrous strands from two papaya cultivars at three developmental stages. We identified carpaine, dehydrocarpaine II, and their derivative alkaloids. Furthermore, we identified two key regulatory genes, *CpNAC82* and *CpHD-Zip ANT2*, associated with alkaloid biosynthesis. Finally, using single-nucleus RNA sequencing technology, we constructed a comprehensive gene expression atlas of papaya fibrous strands and stems, successfully identifying multiple cell types, including epidermal cells, guard cells, parenchyma cells, and phloem cells. Epidermal and phloem cells serve as the primary sites of alkaloid metabolism in papaya. These findings provide new insights into the molecular mechanisms of bitterness in papaya’s fibrous strands and yield genomic resources for improving fruit quality in papaya.

## 1. Introduction

Papaya (*Carica papaya* L.), belonging to the family Caricaceae, is one of the most nutritious and valuable fruits in tropical and subtropical regions of the world. It provides a wide range of phytochemicals, including polysaccharides, glycosides, enzymes, flavonoids, vitamins, and steroids [1,2]. Papaya originates in Southern Mexico and Central America [3]. It is widely cultivated for both its edible fruit and the protease enzyme papain, which has versatile applications in food, medicine, and as an industrial raw material. Papaya fruit can be consumed both in its unripe and ripe stages. Ripe papaya can be peeled and deseeded to be eaten as a fruit, or it can be processed into products such as beverages, jams, jellies, papaya powder, and dried fruit [4,5]. Unripe papaya can be cooked and used in various dishes, including pickles and chutneys. In industrial production, the fibrous strands in papaya fruit impart a bitter taste, are difficult to remove, and tend to adhere to the flesh, significantly compromising the flavor of the fruit.

Bitterness is one of the five basic tastes and significantly influences the flavor of fruits and vegetables. The bitter compounds in plants mainly include phenolic compounds, alkaloids, terpenoids, amino acids, and peptides, as well as glucosinolates [6,7]. Plant phenolic compounds are a class of compounds with multiple hydroxyl groups and are an important source of bitterness. These bitter phenolic compounds are generally categorized into flavonoids, phenolic acids, coumarins, and tannins, including compounds such as quercetin, catechin, epicatechin, and naringin [8,9]. Limonin is a highly oxidized tetracyclic triterpenoid compound and is one of the bitter substances found in citrus fruits [10]. Cucurbitacin is the main source of bitterness in the fruits of cucurbitaceous crops [11]. Glucosinolates, which are widely found in cruciferous plants, are important secondary metabolites, and their degradation products are one of the main sources of bitterness in these plants. For example, progoitrin and glucobrassicin are bitter compounds found in broccoli, Brussels sprouts, and cabbage [12,13]. Alkaloids are widely distributed in plants and play a crucial role in their defense mechanisms, helping plants resist pests and diseases. They typically have a bitter taste, and the intensity of the bitterness is related to the strength of their alkalinity. For example, the steroidal glycoalkaloid in potatoes, piperine in pepper, and caffeine in tea all have a bitter taste [14,15,16].

Papaya leaves contain a wealth of phytochemicals, including alkaloids, enzymes, flavonoids, vitamins, glycosides, amino acids, and other compounds. Canini, et al. [17] used gas chromatography–mass spectrometry (GC-MS) to detect phenolic metabolites in papaya leaves, and during qualitative analysis, identified and characterized compounds such as protocatechuic acid, caffeic acid, p-coumaric acid, chlorogenic acid, quercetin, and kaempferol. Julianti, et al. [18], through high-performance liquid chromatography, successfully identified piperidine alkaloids in papaya leaves, which demonstrated high anti-plasmodial activity. Papaya seeds have been identified as a rich source of thioglucosides and benzyl isothiocyanate [19]. Hiraga, et al. [20] used high-performance liquid chromatography–mass spectrometry to reveal the comprehensive metabolites in both immature and mature papaya fruit skins and flesh. They found that papaya alkaloids such as carpaine, dehydrocarpaine I, and dehydrocarpaine II were mainly present in the fruit skin, with almost no detection in the flesh. Lieb et al. [21] utilized GC-MS to characterize a wide range of aroma-related volatile compounds in papaya fruit, including terpenes, esters, alcohols, and aldehydes. Metabolite analysis of various parts of the papaya plant revealed that the most reported bitter compounds in papaya are primarily found in the leaves, including alkaloids, quercetin and kaempferol. Papaya leaves contain three main alkaloids: carpaine, dehydrocarpaine I, and dehydrocarpaine II [22,23]. Carpaine is one of the first major alkaloids discovered in papaya leaves, found in all the green parts of the plant and its seeds, and it has a strong bitter taste [24]. Carpaine can be extracted from fruits and seeds, with leaves being a particularly rich source [2,25]. Subsequently, Tang [23] isolated two novel piperidine alkaloids, dehydrocarpaine I and dehydrocarpaine II, from papaya leaves and elucidated their structures. However, the compounds in the fruit’s fibrous strands of papaya have not yet been deciphered.

The metabolite composition of fruits is highly complex and can change continuously under the influence of various factors. These factors include fruit variety, ripeness, growth conditions, and post-harvest storage methods, all of which can impact the taste of the fruit. Among these, ripeness is one of the key factors affecting the bitterness of the fruit. In grapefruit, the relative content of limonin decreases with increasing fruit maturity. In young fruits of *Citrus reticulata* ‘Hong Mei Ren’, the content of limonin shows an initial increase followed by a decline during the growth period [26,27]. However, the bitter compounds in papaya fibrous strands and their accumulation dynamics during fruit development remain unclear. Papaya fruit typically reaches full maturity at approximately 150 days after pollination and enters the color-break stage at around 115 days. Therefore, 60, 80, and 100 days after pollination were selected as sampling points in this study, as these stages represent key phases of fruit development prior to the onset of ripening, during which significant changes in metabolite accumulation and transformation are expected.

Weighted gene co-expression network analysis (WGCNA) is a powerful and widely used method that integrates metabolomics and transcriptomics to identify genes associated with key metabolites, further elucidating their regulatory mechanisms [28,29]. It has been used to identify two key enzymes involved in the synthesis of lysine-derived piperidine alkaloids in black pepper (*Piper nigrum*). With the ability to resolve gene expression at single-cell resolution, single-cell transcriptomic technologies have emerged as powerful tools for uncovering the regulatory specificity among individual cells. Their application has rapidly expanded in plant research, including in model species such as *Arabidopsis thaliana* and crop species such as maize, rice, and tomato. For plant tissues such as flowers, stems, fruits, and seeds—where it is often challenging to isolate intact protoplasts—researchers commonly employ single-nucleus transcriptomics, using isolated nuclei as a substitute for whole cells in transcriptome analysis.

‘Zhongbai’ is a widely cultivated papaya variety, optimized through years of commercialization and breeding to meet consumer taste preferences. In contrast, AU9 is an improved but unreleased variety, not yet commercially cultivated, and retains a more original, less desirable flavor profile. The flesh of AU9 is notably more bitter than that of ‘Zhongbai’, highlighting a distinct flavor difference between the two varieties. Therefore, fibrous strands from AU9 and ‘Zhongbai’ papaya fruits at 60, 80, and 100 days of growth were subjected to transcriptomic and metabolomic analyses, combined with single-nucleus transcriptomics of the fibrous strands and stems, to identify key bitter compounds in papaya fruit fibrous strands and the genes involved in their biosynthesis. This study provides valuable insights into the biosynthetic pathways of papaya alkaloids and contributes to the development of papaya cultivars with improved taste and economic value.

## 2. Results

### 2.1. Metabolomics Data Analysis

#### 2.1.1. Metabolite Detection Methods and Quality Assessment

**Figure 1:**
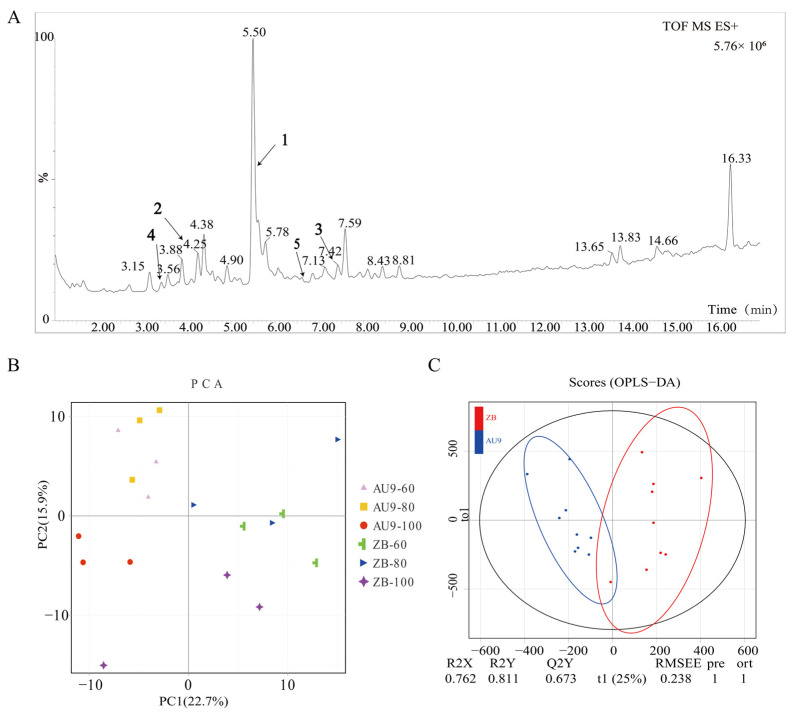
Metabolomic analysis of papaya fibrous strands. (**A**) Total ion chromatogram (TIC) of fibrous strands. The Y-axis represents relative abundance, with each tick corresponding to 10%. (**B**) Principal component analysis (PCA) plot of metabolites in ‘Zhongbai’ and AU9. (**C**) Orthogonal partial least squares discriminant analysis (OPLS-DA) of metabolites in ‘Zhongbai’ and AU9. Note: The numbers 1–5 indicated by arrows in (**A**) correspond to the compound numbers listed in Table 1.

To identify the bitter compounds in the fibrous strands of papaya fruit, we first conducted an untargeted metabolomic analysis in both positive and negative ion modes. The total ion chromatogram (TIC) indicated that more types of compounds were detected in positive ion mode, and the response of alkaloid compounds was higher (Supplementary Figure S1). Therefore, we used liquid chromatography–mass spectrometer (LC-MS) equipment in positive ion mode to analyze the metabolites in the fibrous strands of dioecious AU9 and gynodioecious ‘Zhongbai’ papaya fruits at 60, 80, and 100 days after pollination (Figure 1A). Most of the metabolites were effectively detected within 16 min, and exhibited strong analytical signals, high peak capacity, and good retention time reproducibility. The principal component analysis (PCA) indicated a significant metabolic variance between the 100-day stage and the other two stages (Figure 1B). Orthogonal partial least squares discriminant analysis (OPLS-DA) further revealed significant differences between ‘Zhongbai’ and AU9 (Figure 1C).

**Table 1:** Alkaloid analysis in fruit fibrous strands at different days.

Compound	Molecular Formula	Retention Time	Adducts	*m*/*z*
1	C_14_H_25_NO_3_	5.49	[M+H]^+^	256.1914
2	C_20_H_35_NO_8_	4.26	[M+H]^+^	418.2397
3	C_28_H_46_N_2_O_4_	7.41	[M+H]^+^	475.3530
4	C_14_H_25_NO_4_	3.42	[M+H]^+^	272.1847
5	C_28_H_50_N_2_O_4_	6.71	[2M+H]^+^	240.1963

#### 2.1.2. Identification of Metabolites

After removing redundant peaks and those with a relative abundance below 100, a total of 172 metabolites were detected for subsequent statistical analysis (Supplementary Table S1). These metabolites are categorized as follows: 16 phenolic compounds, 15 organic acids and their derivatives, 19 heterocyclic compounds, 14 alkaloids, 14 glycoside compounds, 13 amino acids and derivatives, nine benzene and substituted derivatives, three sugars, five nucleotides, and derivates, six lipids, and six organosulfur compounds. Based on database annotations and a review of the literature, the peaks in the TIC were identified in descending order of intensity. Several high-abundance compounds were identified as previously reported bitter alkaloids in papaya, including carpaine (compound 5), dehydrocarpaine II (compound 3), and their derivatives (compounds 1, 2, and 4) (Figure 1A and Table 1). The structural identification details are presented below:

Compound 1 showed a precursor ion at *m*/*z* 256.1916 [M+H]^+^ (Supplementary Figure S2C) with a retention time of 5.49 min. The predicted molecular formula was C_14_H_25_NO_3_, which was two hydrogen atoms fewer than that of carpamic acid [18]. This was consistent with the MS/MS fragmentation pattern of the measured dehydrocarpaine II (*m*/*z* 238.19 and *m*/*z* 220.18). We hypothesized that this compound was the precursor of dehydrocarpaine II.

Compound 2 displayed a precursor ion at *m*/*z* 418.2434 [M+H]^+^ (Supplementary Figure S2D) with a retention time of 4.26 min. The predicted molecular formula was C_20_H_35_NO_8_. It differed from compound 1 by C_6_H_10_O_5_, and the MS/MS fragments of compound 2 at *m*/*z* 256.1917 and *m*/*z* 238.1808 were consistent with the MS/MS fragments of compound 1. We hypothesized that it might be a glycoside derivative of compound 1, a putative carpaine derivative.

Compound 3, with a retention time of 7.41 min, exhibited a precursor ion at *m*/*z* 475.3529 [M+H]^+^ and a multivalent ion at *m*/*z* 238.1803 [2M+H]^+^ (Supplementary Figure S2A). The predicted molecular formula was C_28_H_46_N_2_O_4_. The mass of this compound corresponded to that of dehydrocarpaine II, as reported previously.

Compound 4, with a retention time of 3.42 min, exhibited a precursor ion at *m*/*z* 272.1860 [M+H] ^+^ (Supplementary Figure S2E), with a predicted molecular formula of C_14_H_25_NO_4_. It differed from carpamic acid by one oxygen atom. The MS/MS fragmentation patterns at *m*/*z* 254.1757 and *m*/*z* 236.1649 were like the fragment ion pattern of compound 1, a putative carpaine derivative.

The half-ion of compound 5 was detected at *m*/*z* 240.1963 [2M+H]^+^ (Supplementary Figure S2B), with fragment ions at *m*/*z* 222.1852. Based on previous studies, this compound was presumed to be carpaine.

In summary, bitter papaya alkaloids, including carpaine, dehydrocarpaine II, and their derivatives, were identified in papaya fibrous strands. To further examine their dynamic changes during fruit fibrous strands development, the relative abundance of alkaloids was analyzed across different stages. The results (Supplementary Figure S3) showed that alkaloid levels reached their lowest point at 100 days in both cultivars. However, the patterns of change differed between the two cultivars. In AU9, alkaloid content continuously decreased from 60 to 100 days, whereas in ‘Zhongbai’, it first increased and then decreased over the same period.

### 2.2. RNA-Seq Data Analysis

**Figure 2:**
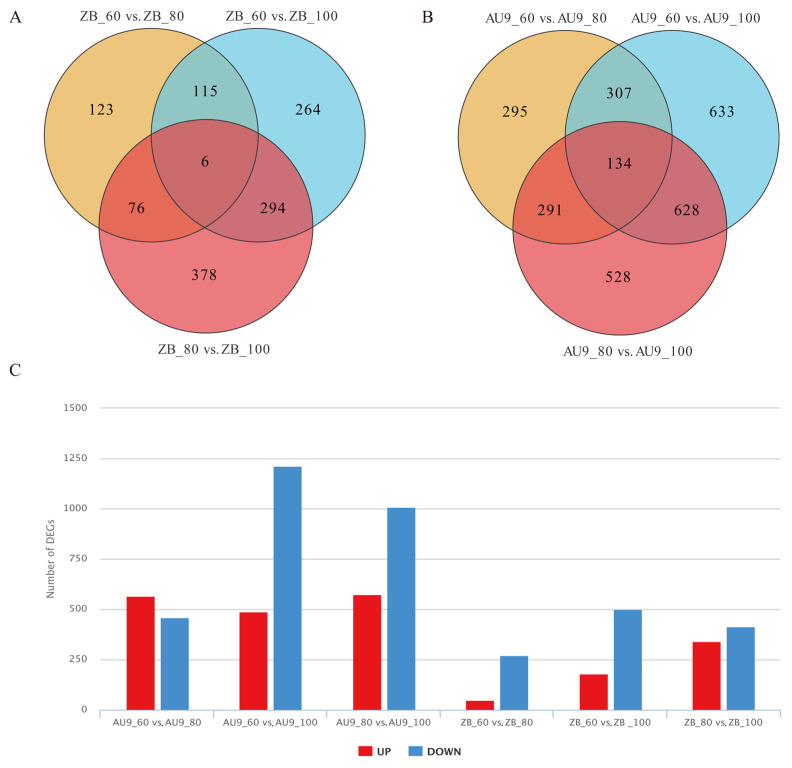
Differential gene expression analysis of ‘Zhongbai’ and AU9 at three developmental stages. (**A**) Venn diagram of differentially expressed genes (DEGs) in various comparison groups (ZB_60 vs. ZB_80, ZB_60 vs. ZB_100, and ZB_80 vs. ZB_100) of papaya fibrous strands in ‘Zhongbai’. (**B**) Venn diagram of DEGs in various comparison groups (AU9_60 vs. AU9_80, AU9_60 vs. AU9_100, and AU9_80 vs. AU9_100) of papaya fibrous strands in AU9. (**C**) Bar chart of upregulated and downregulated DEGs in different comparison groups. Note: ZB represents ‘Zhongbai’, numbers (60, 80, and 100) indicate days after pollination of papaya fibrous strands. For example, ZB_60 vs. ZB_80 represents a comparison of gene expressions in fibrous strands of ‘Zhongbai’ papaya between 60 and 80 days after pollination.

Transcriptome analysis of ‘Zhongbai’ and AU9 at three developmental stages (60, 80, and 100 days) was performed using RNA sequencing (RNA-seq). The transcriptome data of fruit fibrous strands from AU9 and ‘Zhongbai’ papaya at 60, 80, and 100 days were compared to identify differentially expressed genes (DEGs). The results are shown in Figure 2; compared with the cultivar ‘Zhongbai’, the improved but unreleased AU9 exhibited a significantly higher number of DEGs throughout the papaya fiber development cycle. As the number of days of papaya fruit development increased, the number of downregulated DEGs in the fibrous strands also continued to rise. Both ‘Zhongbai’ and AU9 showed a marked decline in alkaloid levels during the 60–100-day and 80–100-day stages. These observations suggest that some of the downregulated genes during these stages may be involved in or associated with alkaloid metabolic pathways in papaya fibrous strands.

### 2.3. WGCNA 

#### 2.3.1. Association Analysis of Papaya Alkaloid Metabolites with Gene Co-Expression Modules

**Figure 3:**
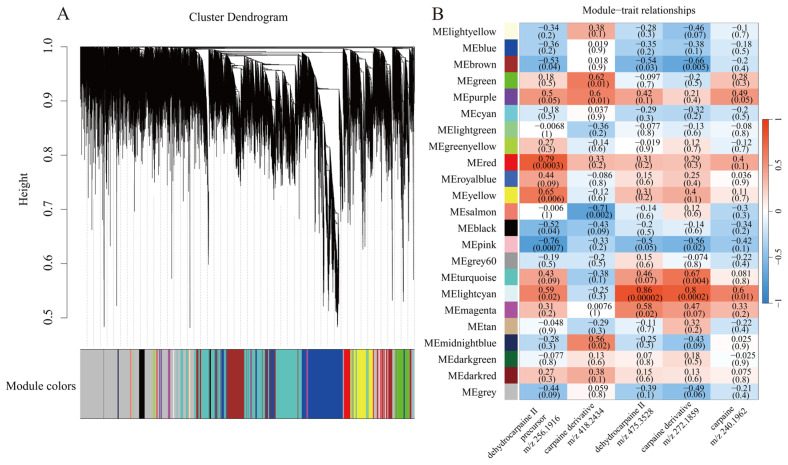
Hierarchical clustering dendrogram (**A**) and heatmap of correlations between gene modules and metabolites (**B**). The correlation coefficients between alkaloid compounds and each module are represented by the data and color in each cell (red indicates a positive correlation, blue indicates a negative correlation).

To identify genes associated with the synthesis of alkaloids and their derivatives, we performed weighted gene co-expression network analysis (WGCNA) on all genes and assigned 16955 genes to 23 co-expression modules, each represented by a distinct color (Figure 3A). The genes in the lightcyan module were strongly correlated with dehydrocarpaine II (*r* = 0.86, *p* = 2 × 10^−5^), carpaine (*r* = 0.6, *p* = 0.01), and carpaine derivatives (*r* = 0.8, *p* = 2 × 10^−4^), indicating that these genes play a key role in alkaloid synthesis. The precursor of dehydrocarpaine II showed a strong correlation with genes in the red (*r* = 0.79, *p* = 3 × 10^−4^) and pink (*r* = −0.76, *p* = 7 × 10^−4^) modules, indicating their importance in the formation of the piperidine ring in papaya alkaloids (Figure 3B). The number of genes in each module is shown in Table 2, ranging from a minimum of 30 to a maximum of 3718 genes. Genes assigned to the grey module did not meet the criteria for module assignment and were therefore excluded from subsequent analyses.

#### 2.3.2. Identification of Transcription Factors in Key Modules

KEGG analysis was performed on genes in the red, pink, and lightcyan modules to identify potential genes involved in the biosynthetic pathways of alkaloids in papaya. The module genes were primarily enriched in plant defense-related pathways, including plant hormone signal transduction (ko04075) and the MAPK signaling pathway–plant (ko04016). In addition, several amino acid metabolic pathways were significantly enriched, such as glycine, serine and threonine metabolism (ko00260), phenylalanine, tyrosine and tryptophan biosynthesis (ko00400), histidine metabolism (ko00340), and cysteine and methionine metabolism (ko00270) (Supplementary Figure S4). Since the biosynthesis of plant alkaloids is largely derived from amino acid metabolism and plays essential roles in plant defense, growth regulation, and overall metabolism, the genes enriched in these pathways are likely associated with alkaloid metabolism in papaya.

**Figure 4:**
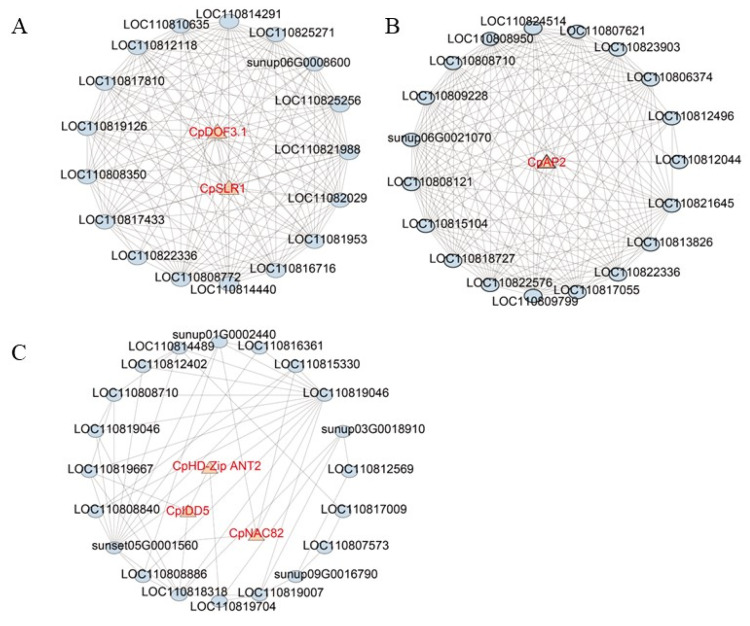
Top 20 hub gene regulatory networks in pink (**A**), red (**B**) and lightcyan (**C**) modules. Triangles represent transcription factors, and ellipses represent regulatory genes.

We constructed interaction networks for the pink, red, and lightcyan modules to identify key genes. Based on their connectivity, the top 20 genes were designated as hub genes. In key pathways, hub genes were highly co-expressed with other genes. In the lightcyan module, we identified transcription factors *LOC110816152* (*CpNAC82*), *LOC110819435* (*CpHD-Zip ANT2*), and *LOC110823752* (*CpIDD5*), which may play a role in regulating the biosynthesis of papaya alkaloids. In the pink and red modules, we identified transcription factors *LOC110812717* (*CpSLR1*), *LOC110808866* (*CpDOF3.1*), and *LOC110809095* (*CpAP2*), which may be involved in regulating the synthesis of dehydrocarpaine II precursors (Figure 4).

### 2.4. qRT-PCR

**Figure 5:**
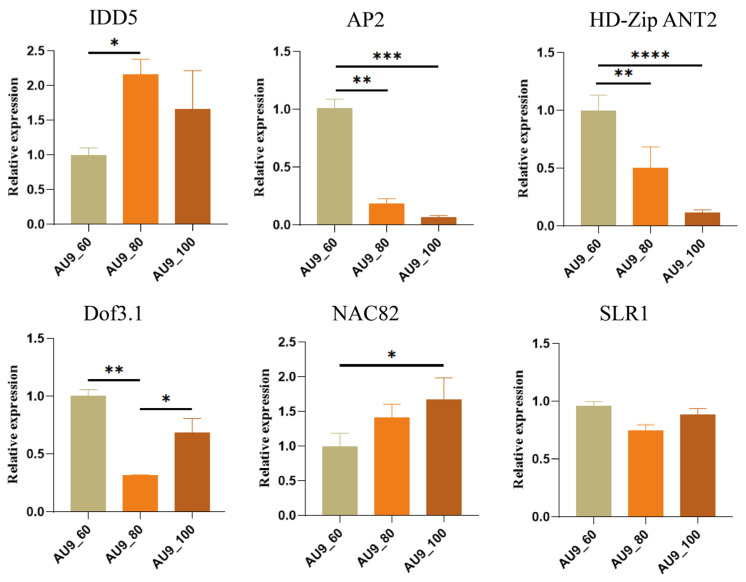
Validation by quantitative real-time PCR. Error bars indicate the standard error of three biological replicates. Significance levels: * *p* < 0.05, ** *p* < 0.01, *** *p* < 0.001, **** *p* < 0.0001.

The transcriptome data for AU9 exhibited good reproducibility, with no outliers. To verify the reliability of the transcriptome data and provide a foundation for future functional validation of the genes, we selected six genes for real-time fluorescence quantitative PCR (qRT-PCR) validation in AU9. As illustrated in Figure 5, during the 60- to 100-day fibrous strands development period, the expression of *CpHD-Zip ANT2* declined in tandem with alkaloid levels, while the *CpNAC82* showed an inverse pattern, with increased expression accompanying the reduction in alkaloid content.

### 2.5. Single-Nucleus Transcriptome Analysis

#### 2.5.1. Basic Analysis of snRNA-Seq Data from Papaya Fibrous Strands and Stem

**Figure 6:**
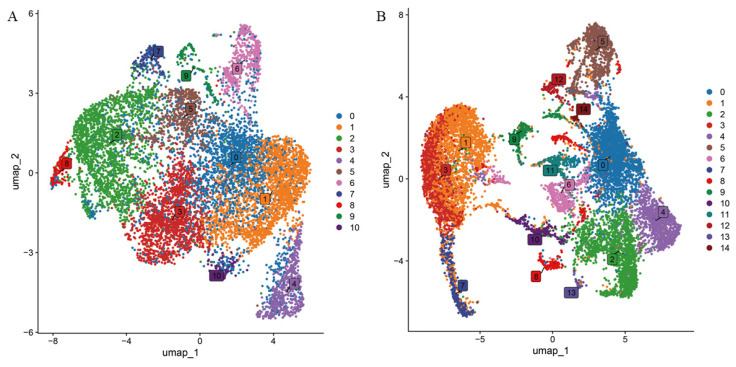
Cellular heterogeneity in the fibrous strands and stems of papaya. (**A**) Visualization of fibrous strands cell clusters using the UMAP algorithm. (**B**) Visualization of stem cell clusters using the UMAP algorithm. Each dot denotes a nucleus. Point colors correspond to distinct cell clusters.

To further investigate the spatial localization patterns and expression differences of genes involved in the regulation of alkaloid biosynthesis in papaya, single-nucleus RNA sequencing (snRNA-seq) was performed on two tissues: fibrous strands and stem. After quality control at both the cellular and gene levels, 11,105 nuclei and 20,145 genes were retained from the fibrous strands, and 11,524 nuclei and 20,116 genes from the stem. Based on the top 2000 highly variable genes, dimensionality reduction and clustering were performed separately for the fibrous strands and the stem. Uniform Manifold Approximation and Projection (UMAP) visualization identified 11 clusters in the fibrous strands and 15 in the stem (Figure 6).

#### 2.5.2. Identification of Cell Types with Marker Genes

**Figure 7:**
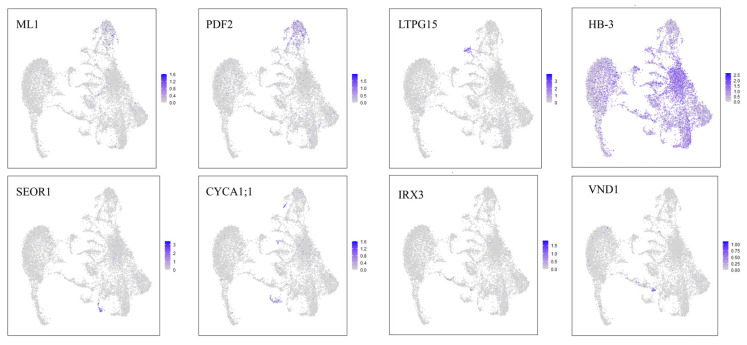
UMAP visualization of marker genes in papaya stem. Each dot denotes a nucleus.

In *Arabidopsis*, the epidermis-related marker genes *FIDDLEHEAD* (*FDH*), *MERISTEM LAYER 1* (*ML1*), and Protodermal Factor 2 (PDF2) [30] were found to be specifically and highly expressed in cluster 5 of the papaya stem, which was annotated as epidermal cells. Cluster 13 was annotated as phloem cells (PhCs) based on the phloem-specific expression of *ALTERED PHLOEM DEVELOPMENT* (*APL*) and several members of the PHLOEM PROTEIN 2 (PP2) gene family, including *PP2-A1*, *PP2-A4*, and *PP2-A10*. Cluster 14 was annotated as guard cells (GCs) based on the high and specific expression of the multi-transmembrane protein gene *SLOW ANION CHANNEL-ASSOCIATED 1* (*SLAC1*) [31], which is known to be guard cell-specific in Arabidopsis. *SLAC1* primarily regulates the maturation and structural formation of guard cells and can thus serve as a marker gene for this cell type. Guard cell marker genes, including *FAMA* [32] and *ALUMINUM-ACTIVATED MALATE TRANSPORTER 12* (*ALMT12*), exhibited specific expression exclusively within this cluster. Clusters 0, 2, and 12 were annotated as cortex cells. Marker genes that are highly expressed in the root tip cortex cells of *Arabidopsis*, including *NUCLEOLIN 1* (*NUC*), *HOMEOBOX PROTEIN 3 (HB-3*), and *GLYCOSYLPHOSPHATIDYLINOSITOL-ANCHORED LIPID TRANSFER PROTEIN 15* (*LTPG15*) [33], also exhibited markedly high expression in these clusters. Clusters 10 and 11 were annotated as xylem cells, showing enriched expression of several key regulatory genes closely associated with xylem differentiation, including *VASCULAR-RELATED NAC-DOMAIN PROTEIN 1* (*VND1*) [34], *SECONDARY WALL-ASSOCIATED NAC DOMAIN PROTEIN 1* (*SND1*), *MONOPTEROS* (*MP*), and *IRREGULAR XYLEM 3* (*IRX3*) [35], indicating that they possess the typical characteristics of xylem development. Clusters 7 and 8 were annotated as meristematic cells (MCs), exhibiting significant expression of multiple cell cycle-related genes, including *HISTONE H4* (*HIS4*), *TSO2*, *DEVELOPMENT-RELATED PCG TARGET IN THE APEX (DPA)*, *STIGMA/STYLE CELL-CYCLE INHIBITOR 1* (*SCI1*), and *MERISTEMATIC RECEPTOR-LIKE KINASE* (*MRLK*), indicating that they possess the typical characteristics of meristematic cells. In addition, DEGs in clusters 3 and 4 were mainly enriched in cysteine-type endopeptidase activity, which is highly expressed during fruit ripening and senescence, as well as in responses to water, heat, and cold. The results of GO enrichment analysis are shown in Supplementary Figure S5. These features suggest that these clusters represent parenchyma cells. Marker genes used for cell-type identification are provided in Supplementary Table S4. Figure 7 shows the UMAP plot of papaya homologous marker genes across different cell types. In summary, seven cell types were identified in the papaya stem: cortex cells (CCs), epidermis cells (ECs), parenchyma cells (PaCs), guard cells (GCs), phloem cells (PhCs), meristematic cells (MCs), and xylem cells (XCs).

The phloem-related genes *APL* and *PP2-A1* were specifically expressed in clusters 6, 9 and 10 of the papaya fibrous strands. Meanwhile, DEGs in cluster 6 were enriched in the regulation of the vascular endothelial growth factor receptor signaling pathway. Therefore, clusters 6, 9, and 10 in the fibrous strands were annotated as vascular cells (Supplementary Figure S6). The DEGs in cluster 1 showed GO enrichment consistent with those of stem clusters 3 and 4; therefore, cluster 1 was annotated as parenchyma cells. The DEGs of cluster 5 were significantly enriched in GO terms related to sclerenchyma cell differentiation; thus, this cluster was annotated as sclerenchyma cells. The results of GO enrichment analysis are shown in Supplementary Figure S5. In summary, some of the cell types within the vascular bundles were identified as parenchyma cells (PaCs), vascular cells (VCs), and sclerenchyma cells (SCs).

#### 2.5.3. Expression of Alkaloid Biosynthesis-Related Genes in Papaya

**Figure 8:**
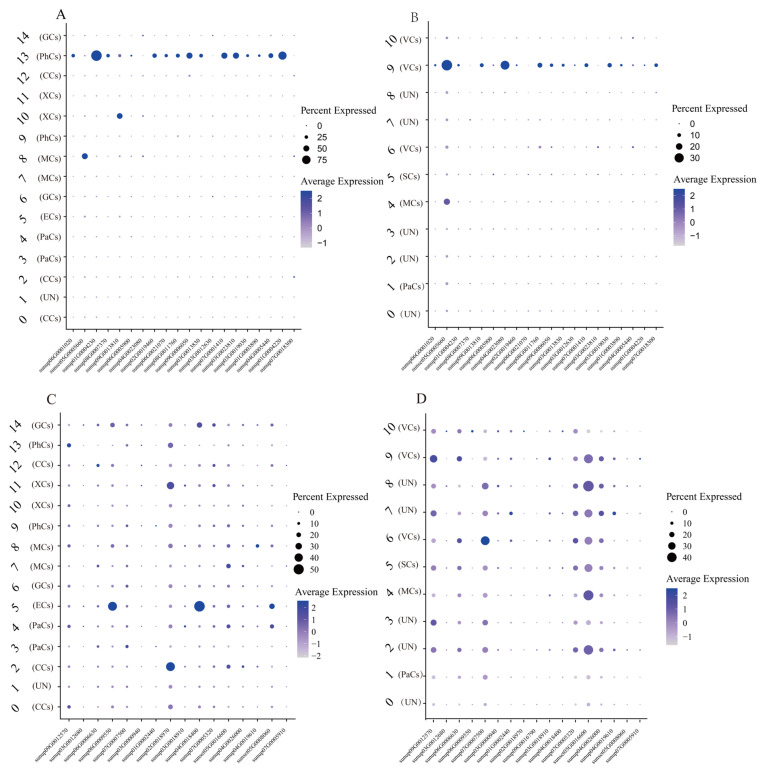
Expression patterns of red module genes across different cell types in stem (**A**) and fibrous strands (**B**), and expression patterns of lightcyan module genes in stem (**C**) and fibrous strands (**D**). Note: The X-axis represents the top 20 most-connected genes from the red and lightcyan modules identified by WGCNA, and the Y-axis represents different cell types in stem and fibrous strands. CCs, cortex cells; PaCs, parenchyma cells; ECs, epidermis cells; GCs, guard cells; MCs, meristematic cells; XCs, xylem cells; VCs, vascular cells; PhCs, phloem cells; UN, unknown.

Based on the WGCNA results, the top 20 most-connected genes in the red module, which exhibited the highest correlation with the precursor of dehydrocarpaine II (*r* = 0.79, *p* = 0.0003), were selected for expression analysis. As shown in Figure 8A,B, genes in this module were almost exclusively expressed in phloem cells. We further analyzed the top 20 most-connected genes in the lightcyan module, which showed the highest correlation with the bitter compound dehydrocarpaone II in papaya (*r* = 0.86, *p* = 2 × 10^−5^). This module contained a total of 49 genes, most of which displayed low expression levels, while the highly expressed genes were predominantly detected in the epidermal cells of the papaya stem. Taken together, these findings suggest that both epidermal and phloem cells serve as major sites for the alkaloid metabolic pathway underlying papaya bitterness. Given that the vascular cells in papaya vasculature contain phloem cells, this cell type is likely the primary contributor to the bitterness of the vasculature.

#### 2.5.4. Differentiation Trajectory of Epidermal Cells and Vascular Cells

Trajectory analysis facilitates the exploration of dynamic gene expression patterns in specific cell types and provides an intuitive visualization of cellular differentiation through dimensionality reduction. To validate the dynamic expression changes of key genes during the developmental trajectories of epidermal and vascular cells, we applied Monocle2 to perform pseudo time analysis and explore their continuous differentiation paths. Epidermal cells (cluster 5) from the papaya stem were selected for trajectory analysis, and genes from the lightcyan module that were highly expressed in epidermal cells were visualized to investigate their expression patterns along the epidermal cell differentiation trajectory. As shown in Supplementary Figure S7, genes associated with alkaloid metabolism maintained consistently high expression throughout the entire developmental trajectory of the epidermal cells, further supporting the critical role of these cells in the synthesis and accumulation of alkaloid compounds in papaya.

**Figure 9:**
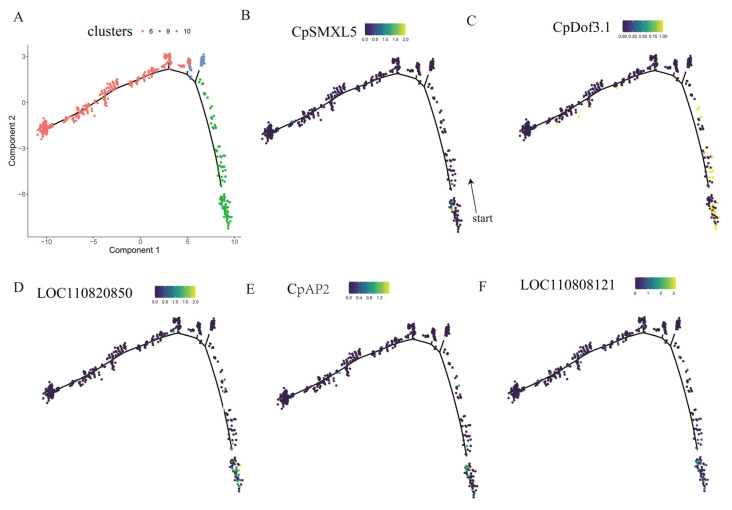
Differentiation trajectory of vascular cells in papaya fibrous strands. (**A**): Trajectory visualization of vascular cells (cluster 6, 9, and 10). Component 1 and Component 2 represent the reduced dimensions obtained from dimensionality reduction. Each dot denotes a nucleus. (**B**): *CpSMXL5* expression in vascular cells. (**C**): Cp Dof3.1 expression in vascular cells. (**D**–**F**): Expression pattern of hub genes in the red module within vascular cells. Note (**B**–**F**): The color gradient of the points indicates the relative expression level of the indicated gene in each nucleus.

Cell clusters annotated as vascular cells from the papaya fibrous strands (cluster 6, 9 and 10) were selected to construct their developmental trajectory (Figure 9A). The *SMXL5* [36] gene is one of the marker genes for early phloem cell identification in *Arabidopsis thaliana*. Based on its highest homolog in papaya, this gene was used as the starting marker for tracing the phloem differentiation trajectory in papaya (Figure 9B). Genes with high connectivity in the red module (Figure 9D–F) exhibited elevated expression levels during the early developmental stages of phloem cells. *CpDof3.1* (Figure 9C) showed consistent expression during the entire course of phloem development. This suggests that phloem cells serve as the primary metabolic site for alkaloid-related genes in papaya, and that the early developmental stage of the phloem represents a critical period for this metabolism.

## 3. Discussion

Fruits are one of the important sources of essential nutrients for human health, and their flavor is crucial to consumers. Most fruits have a primarily sweet flavor; some also have a bitter taste, which can reduce palatability and affect commercial value. Hiraga [20], through a comparative analysis of metabolites in papaya peel and pulp, found that the alkaloids carpaine, dehydrocarpaine I, and dehydrocarpaine II were all present in the peel, while their levels in the pulp were negligible or undetectable. Therefore, it can be inferred that the alkaloids enriched in papaya fibrous strands may be one of the main contributors to their bitterness. However, in addition to alkaloids, untargeted metabolomic analysis detected many unknown compounds, suggesting that the composition of bitter compounds in papaya fibrous strands is more complex than initially described. Moreover, the dynamic accumulation patterns of alkaloids differ between the two cultivars. For instance, the most abundant alkaloid (Supplementary Table S1) shows similar levels in ‘Zhongbai’ and AU9 at 60 days, but at 80 and 100 days, its content is higher in ‘Zhongbai’ than in AU9. In contrast, the second most abundant alkaloid exhibits higher levels in AU9 than in ‘Zhongbai’ across all three time points. These observations indicate that bitterness is not solely determined by the abundance of individual alkaloids but may also result from interactions among multiple compounds and their temporal dynamics during fruit development. Future studies are needed to clarify the contributions of different metabolites to papaya bitterness.

WGCNA identified 49 genes within the lightcyan module that showed strong correlations with carpaine and dehydrocarpaine II levels. qRT-PCR revealed that two key transcription factors, *CpNAC82* and *CpHD-Zip ANT2*, may be involved in the biosynthesis of papaya alkaloids. During the 60- to 100-day period in AU9, the expression level of the *CpHD-Zip ANT2* transcription factor continuously decreased, consistent with the trends observed in both the transcriptome data and the decline in alkaloid content. In contrast, the expression of the *CpNAC82* transcription factor was opposite to that of the alkaloid content. The NAC transcription factor family is one of the most important families of transcription factors in plants, playing a critical role in responses to abiotic stresses such as drought, salinity, low temperature, and diseases. NAC transcription factor family members actively regulate plant disease resistance by directly activating the expression of lignin-related genes as validated by overexpression and knockout experiments [37]. Recently, a transcription factor—*NAC90*—was identified as a negative regulator of the biosynthetic pathway of *N*-hydroxy pipecolic acid (NHP). The negative regulatory role of *NAC90* in disease resistance responses was entirely mediated through the biosynthesis of NHP. Upon pathogen infection, *NAC90* formed a complex with other family members to jointly suppress NHP biosynthesis, thus maintaining a balance between plant growth and defense [38]. HD-ZIP transcription factors are an important class of plant transcription factors that regulate plant responses to biotic stresses. Wang, et al. [39] identified 32 HD-ZIP transcription factor members across the whole genome of *Ophiorrhiza pumila*. Based on transcriptome data, co-expression analysis, and dual-luciferase reporter assays, they identified two HD-ZIP transcription factors that activate the expression of genes involved in camptothecin biosynthesis. However, the specific regulatory roles of *CpNAC* and *CpHD-Zip ANT2* transcription factors in the alkaloid metabolism of papaya remain to be further explored.

Gypenosides are the primary bitter-tasting active constituents of *Gynostemma pentaphyllum*. Li [40] constructed cell transcriptomic atlases of leaf and shoot tips, revealing that genes involved in gypenoside biosynthesis are predominantly expressed in epidermal and xylem cells. In *Catharanthus roseus* leaves, multiple monoterpenoid indole alkaloids are present. Li [41] established a protoplast isolation system suitable for *C. roseus* leaves and performed scRNA-seq analysis, systematically characterizing the expression patterns of key genes in the monoterpenoid indole alkaloid biosynthetic pathway across distinct cell types. Their findings demonstrated that alkaloid biosynthesis initiates in phloem parenchyma cells and culminates in epidermal cells, where the synthesis of bisindole alkaloids is completed. Building on these findings, scRNA-seq may provide a powerful approach to further dissect the metabolic pathways in papaya. Building on the WGCNA results, we further performed snRNA-seq on fibrous strands and stem tissues to determine the specific cell types expressing alkaloid-related genes. Our preliminary analysis suggests that epidermal cells and phloem cells serve as the primary sites of alkaloid metabolism in papaya. This finding not only provides important insights into the molecular mechanisms underlying bitterness formation in papaya but also establishes a theoretical foundation for subsequent functional gene validation and the breeding of high-quality papaya cultivars.

## 4. Materials and Methods

### 4.1. Plant Materials and Growth Conditions

In this study, the papaya cultivars used for transcriptome and metabolome sequencing were gynodioecious ‘Zhongbai’ and dioecious AU9, both grown at a papaya plantation in Yongchun County, Quanzhou, Fujian Province, China (approximately 25.3795° N, 118.2579° E). For each cultivar, fruits that were intact and free of mechanical damage were collected, as shown in Supplementary Figure S8A,B. Fruits were sampled at three developmental stages: 60, 80, and 100 days after pollination. Each fruit was longitudinally cut, the seeds were removed, and the fibrous strands were carefully dissected with a blade (Supplementary Figure S8C). Three biological replicates were collected for each developmental stage, and all fibrous strands samples were stored at −80 °C until further use.

For snRNA-seq, one papaya fibrous strands sample and one papaya stem sample, both from AU9, were used. The fibrous strands sample was obtained from fruits grown at a papaya plantation in Yongchun County, Quanzhou, Fujian Province, China. Three fruits approximately 45 days after pollination were collected; each fruit was longitudinally cut, the seeds were removed, and the fibrous strands were dissected with a blade and pooled. The stem sample was collected from the apical region of approximately 2-year-old AU9 trees located on the campus of Fujian Agriculture and Forestry University, China. Both samples were immediately sent to the sequencing company for snRNA-seq after collection.

### 4.2. Non-Targeted Metabolome Analysis

The fibrous strands samples of ‘Zhongbai’ and AU9 papaya collected at 60 d, 80 d, and 100 days were ground into powder in liquid nitrogen and subsequently freeze-dried under vacuum conditions. About 30 mg of the powder was dissolved in 1 mL of precooled 70% methanol extraction solution, followed by sonication for 20 min, and then centrifuged for 15 min (12,000 r/min, 4 °C). The supernatant was collected and stored in an injection vial for liquid chromatography–mass spectrometry (LC-MS).

LC-MS liquid chromatography conditions: The chromatographic analysis was performed using an Acuquity UPLC HSS T3 column (2.1 × 100 mm, 1.8 µm). The mobile phases consisted of 0.1% formic acid in water (A) and acetonitrile with 0.1% formic acid (B). The column temperature was maintained at 40 °C, with a flow rate of 0.3 mL/min. The injection volume was 1 µL. The gradient elution program was as follows: 0–2 min (99–93% A), 2–13 min (93–60% A), 13–17 min (60–40% A), 17–22 min (40–1% A), and 22–25 min (99–99% A). LC-MS mass spectrometry conditions: The mass spectrometry analysis was conducted using an ESI ion source, operating in both negative (ESI^−^) and positive (ESI^+^) ionization modes. The capillary voltage was set to 1.50 kV, with an MSE ramp energy of 10–40 eV. The scan range was 50–1500 Da. Additional parameters were as follows: ion source temperature, 120 °C; desolvation temperature, 350 °C; desolvation gas flow rate, 800 L/h; cone gas flow rate, 50 L/h; and cone voltage, 60 V.

Baseline filtering, peak alignment, peak area normalization, and data collection were performed using Progenesis QI v2.0. Data analysis and plotting were carried out using MassLynx (v4.2).

### 4.3. Transcriptome Sequencing and Analysis

Total RNA was extracted from papaya fibrous strands using the RNAprep Pure Plant Plus Kit (Polysaccharides & Polyphenolics-rich) (TIANGEN, Beijing, China). Library construction and transcriptome sequencing were performed by Beijing Novogene Bioinformatics Technology Co., Ltd. (Beijing, China) using the Illumina HiSeq™ 2500 platform [42,43]. Three biological replicates were created for each sample, and a total of 18 cDNA libraries were constructed for RNA-seq. Each sample was sequenced to a depth of 3 G, yielding a total of 1.0~1.4 × 10^8^ raw reads. After adapter trimming and the removal of low-quality sequences, high-quality sequences were obtained, ranging from 9.5 × 10^7^ to 1.3 × 10^8^ (Supplementary Table S2).

The raw data from sequencing was filtered using Trimmomatic [44] to remove connectors and low-quality sequences from the sequencing data to obtain high-quality clean data. The filtered high-quality sequences were compared to the papaya SunUp genome (https://doi.org/10.5281/zenodo.19368772) (accessed on 1 April 2026) using Hisat2 [45] software, and normalized by the transcripts per million (TPM) method. DESeq2 [46] was then used to analyze differentially expressed genes (DEGs) between the samples. Screening conditions were |log2 (fold change)| ≥ 1, adjusted *p* value ≤ 0.05. The online website eggNOG-mapper [47] was used to annotate the gene functions, and KO numbers and GO numbers were extracted as background files. DEGs were subjected to GO (Gene Ontology) functional enrichment and KEGG (Kyoto Encyclopedia of Genes and Genomes) pathway enrichment analyses using the OmicShare platform (https://www.omicshare.com/tools/) (accessed on 1 April 2026) [48]. The analyses were performed using OmicShare with its default parameter settings.

### 4.4. Weighted Gene Co-Expression Network Analysis

The construction of weighted gene co-expression network analysis (WGCNA) was implemented using the WGCNA [29] package in R version 4.3.3. We performed TPM normalization on all gene expression data obtained from transcriptome sequencing of AU9 and ‘Zhongbai’ at three stages: 60, 80, and 100 days. Genes with a total TPM sum greater than 1 across all samples were selected for Pearson’s correlation coefficients (PCC) analysis, and different modules were identified. Then, the relative abundance of metabolites measured through untargeted metabolomics was analyzed for correlation with each module using Pearson’s correlation to identify strongly correlated modules. The strong correlation criteria were |PCC| ≥ 0.6 and *p* < 0.01. Finally, the corresponding association networks were visualized using Cytoscape (v3.9.1) [49].

### 4.5. qRT-PCR Validation

The total RNA of the fibrous strands was extracted using the total RNA kit (vazyme, Nanjing, China). The quality and concentration of the RNA were detected by agarose gel electrophoresis and a Nanodrop spectrophotometer. The cDNA was synthesized by a HiScript IV 1st Strand cDNA Synthesis Kit (vazyme, Nanjing, China). Six candidate genes related to alkaloid biosynthesis were randomly selected, and corresponding gene-specific primers were designed (Supplementary Table S3). We selected the 2^−ΔΔCT^ relative quantification method to analyze the relative expression level [50].

### 4.6. Single-Nucleus RNA Sequencing and Analysis

#### 4.6.1. Preparation of Nuclei Suspension from Papaya Protoplasts

Nuclei isolation [51,52] was performed by Gene Denovo (Guangzhou, China).

(1) Buffer preparation: The nuclei isolation buffer (NIB) was thoroughly mixed and filtered through a 0.22 μm filter.

The wash buffer was thoroughly mixed and filtered through a 0.22 µm filter.

(2) Isolation of nuclei: ➀ The samples were minced with a sterile razor blade for 2 min, repeated 2–3 times, until a homogenate was obtained with no large tissue fragments remaining. ➁ The homogenized samples were carefully transferred into centrifuge tubes containing NIB prepared according to the Table 3 and centrifuged at 300× *g* for 1 min. The supernatant was collected into a new centrifuge tube. ➂ The supernatant was passed sequentially through a 70 μm cell strainer into a new 50 mL tube, followed by a 40 μm cell strainer into a new 15 mL tube. ➃ The filtrate was centrifuged at 2000× *g* for 5 min. The supernatant was discarded, and the filtrate was resuspended in wash buffer. ➄ The nuclei suspension was adjusted to a concentration of 1000–2000 nuclei/µL using wash buffer prepared according to the Table 4 and kept on ice until further use.
(3) Fluorescence-activated nuclei sorting: ➀ Flow cytometry sample tubes were pre-cooled to 4 °C. A mixture of 100 μL of nuclei and 900 μL of wash buffer was transferred into a sample tube as an unstained control. A 70 μm nozzle was used with the default pressure set at 20 psi. Based on FSC-A vs. SSC-A, a gate was set to select small particles and exclude large debris. ➁ DAPI was added to the remaining single-nucleus suspension to a final concentration of 10 μM. The stained nuclei suspension was transferred into a flow cytometry sample tube. Another sample tube containing 1 mL of wash buffer was used to collect the sorted nuclei. Nuclei were sorted based on DAPI signal and nuclear size. ➂ The sorted nuclei suspension was centrifuged at 2000× *g* for 5 min at 4 °C. The supernatant was discarded, and the sorted nuclei suspension was resuspended in wash buffer. ➃ Then, 10 μL of the sorted nuclei suspension was stained with DAPI to a final concentration of 10 μM and examined using the DAPI fluorescence channel to assess nuclei quality and count. The collected nuclei were diluted to approximately 1000 nuclei/μL. ➄ The prepared single-nucleus suspension was immediately used for 10x Genomics library preparation.

#### 4.6.2. Library Construction and Sequencing

The library synthesis and RNA sequencing were completed by the Gene Denovo (Guangzhou, China). The indexed sequencing libraries were prepared using Chromium Single Cell 3′ Reagent Kits (V3.1) (10x Genomics, Pleasanton, CA, USA) according to the manufacturer’s instructions. The final Single Cell 3′ Libraries contained the P5 and P7 primers used in the Illumina bridge amplification PCR. The barcoded sequencing libraries were quantified using a standard curve-based qPCR assay (KAPA Biosystems, Wilmington, MA, USA) and Agilent Bioanalyzer 2100 (Agilent, Loveland, CO, USA). Finally, library sequencing was carried out on an Illumina NovaSeq 6000 using paired end multiplexing with a read length of 150 bp.

#### 4.6.3. snRNA-Seq Bioinformatics Analysis

Data preprocessing: The gene–nucleus expression matrix was generated using the Cell Ranger software (v9.0.0). The resulting gene–nucleus expression matrix was subjected to downstream analysis using the Seurat package (version 4.1.1). Filtering criteria for stem sequencing data were as follows: (1) nuclei with nFeature_RNA counts between 500 and 3000; (2) nuclei with nCount_RNA greater than 600 or less than 6000; (3) genes expressed in fewer than three nuclei were removed. Filtering criteria for vascular tissue sequencing data were as follows: (1) nuclei with nFeature_RNA counts between 700 and 5000; (2) nuclei with nCount_RNA greater than 900 or less than 12,000; (3) genes expressed in fewer than three nuclei were removed.

Data normalization and clustering: Data were normalized, centered, and subjected to variable feature selection (variable.features.n = 2000) and PCA (using the RunPCA function with ndims = 50) via SCTransform. Visualization based on the Louvain algorithm (FindNeighbors and FindClusters) and nonlinear dimensionality reduction methods (RunTSNE and RunUMAP) were performed sequentially.

Marker gene analysis: DEGs in each cell cluster were identified using the FindConservedMarkers function in the Seurat package. Cell cluster annotation was performed using two complementary approaches: (1) identification based on previously reported marker genes in plants by comparing highly homologous genes in papaya; and (2) functional annotation through GO enrichment analysis of genes specifically expressed in different clusters.

Trajectory analysis: Trajectory analysis of specific cell types was performed using the Monocle2 package (V2.30.1) [53]. The dispersionTable function was used to calculate gene expression variability across cells. Genes with high average expression levels (mean_expression) were selected to define the developmental trajectory. The trajectory was visualized using the plot_cell_trajectory function, and the starting point of the trajectory was adjusted using the root_state parameter.

## Figures and Tables

**Table 2:** Number of genes in each co-expression module identified by WGCNA based on metabolomic transcriptomic data from papaya fibrous strands at 60, 80, and 100 days.

Module	Number	Module	Number	Module	Number
darkgreen	30	darkred	31	royalblue	33
lightgreen	35	lightgreen	35	grey60	44
lightcyan	49	midnightblue	53	cyan	56
salmon	66	tan	75	greenyellow	98
purple	102	magenta	260	pink	282
black	332	red	450	green	638
yellow	903	brown	2006	blue	2626
turquoise	3718	grey	5033		

**Table 3:** Preparation of nuclei isolation buffer (NIB).

Reagent	Final Concentration	Volume Added
DextranT40	5%	0.5 g
Sucrose (2 M)	0.4 M	2 mL
MgCl_2_ (1 M)	10 mM	100 μL
Dithiothreitol (DTT)	1 mM	10 μL
RNase inhibitor	2 U/μL	50 μL
Triton X-100 (30%)	0.1%	33 μL
Tris-HCl (1 M)	100 mM	1 μL
RNase-free water	/	Up to 10 mL

**Table 4:** Preparation of wash buffer.

Reagent	Final Concentration	Volume Added
PBS	10 mM	1 mL
BSA	1%	500 μL
RNase inhibitor	2 U/μL	50 μL
RNase-free water	/	Up to 10 mL

## Data Availability

The raw RNA sequence data reported in this paper have been deposited in the Genome Sequence Archive [54] in the National Genomics Data Center Members and Partners [55], China National Center for Bioinformation (GSA: CRA026972, CRA028038). The raw metabolomics data reported in this paper have been deposited in the OMIX, China National Center for Bioinformation, Chinese Academy of Sciences (accession no. OMIX010758). The papaya ‘SunUp’ genome data used for transcriptome and snRNA-seq alignment are available on Zenodo (https://doi.org/10.5281/zenodo.19368772).

## Supplementary Materials

The following supporting information can be downloaded at https://www.mdpi.com/article/10.3390/ijms27083438/s1.

## References

[1] Sharma N., Mishra K.P., Chanda S., Bhardwaj V., Tanwar H., Ganju L., Kumar B., Singh S.B. (2019). Evaluation of anti-dengue activity of *Carica papaya* aqueous leaf extract and its role in platelet augmentation. Arch. Virol..

[2] Verma N. (2014). A Brief Study on *Carica papaya*—A review. Int. J. Curr. Trends Pharm. Res..

[3] Teixeira da Silva J., Rashid Z., Duong Tan N., Sivakumar D., Gera A., Souza Junior M., Tennant P. (2007). Papaya (*Carica papaya* L.) biology and biotechnology. Tree For. Sci..

[4] Lal Bajya S., Shankar Bunkar D., Kumar Goyal S., Kumar Singh M., Kumar Paswan V., Lal S., Dhyani P. (2024). Foodomics-based metabolites profiling of the Greek yogurt incorporated with unripened papaya peel powder. Food Chem. Mol. Sci..

[5] Ikram E.H.K., Stanley R., Netzel M., Fanning K. (2015). Phytochemicals of papaya and its traditional health and culinary uses—A review. J. Food Compos. Anal..

[6] Chu X., Zhu W., Li X., Su E., Wang J. (2024). Bitter flavors and bitter compounds in foods: Identification, perception, and reduction techniques. Food Res. Int..

[7] Soares S., Kohl S., Thalmann S., Mateus N., Meyerhof W., De Freitas V. (2013). Different phenolic compounds activate distinct human bitter taste receptors. J. Agric. Food Chem..

[8] Drewnowski A., Gomez-Carneros C. (2000). Bitter taste, phytonutrients, and the consumer: A review. Am. J. Clin. Nutr..

[9] Osakabe N., Shimizu T., Fujii Y., Fushimi T., Calabrese V. (2024). Sensory nutrition and bitterness and astringency of polyphenols. Biomolecules.

[10] Tundis R., Loizzo M.R., Menichini F. (2014). An overview on chemical aspects and potential health benefits of limonoids and their derivatives. Crit. Rev. Food Sci. Nutr..

[11] Shang Y., Ma Y., Zhou Y., Zhang H., Duan L., Chen H., Zeng J., Zhou Q., Wang S., Gu W. (2014). Biosynthesis, regulation, and domestication of bitterness in cucumber. Science.

[12] Carlson D., Daxenbichler M.E., Vanetten H., Kwolek W.F., Williams P.H. (1987). Glucosinolates in crucifer vegetables: Broccoli, brussels sprouts, cauliflower, collards, kale, mustard greens, and kohlrabi. J. Am. Soc. Hortic. Sci..

[13] Wieczorek M.N., Michał W., Marzena S.-Z., Jeleń H.H. (2018). Bitter taste of *Brassica* vegetables: The role of genetic factors, receptors, isothiocyanates, glucosinolates, and flavor context. Crit. Rev. Food Sci. Nutr..

[14] Cárdenas P.D., Sonawane P.D., Heinig U., Bocobza S.E., Burdman S., Aharoni A. (2015). The bitter side of the nightshades: Genomics drives discovery in Solanaceae steroidal alkaloid metabolism. Phytochemistry.

[15] Lv Y., Zhu J., Huang S., Xing X., Zhou S., Yao H., Yang Z., Liu L., Huang S., Miao Y. (2024). Metabolome profiling and transcriptome analysis filling the early crucial missing steps of piperine biosynthesis in *Piper nigrum* L.. Plant J..

[16] Deng S., Zhang G., Olayemi Aluko O., Mo Z., Mao J., Zhang H., Liu X., Ma M., Wang Q., Liu H. (2022). Bitter and astringent substances in green tea: Composition, human perception mechanisms, evaluation methods and factors influencing their formation. Food Res. Int..

[17] Canini A., Alesiani D., D’Arcangelo G., Tagliatesta P. (2007). Gas chromatography–mass spectrometry analysis of phenolic compounds from *Carica papaya* L. leaf. J. Food Compos. Anal..

[18] Julianti T., De Mieri M., Zimmermann S., Ebrahimi S.N., Kaiser M., Neuburger M., Raith M., Brun R., Hamburger M. (2014). HPLC-based activity profiling for antiplasmodial compounds in the traditional Indonesian medicinal plant *Carica papaya* L.. J. Ethnopharmacol..

[19] Nakamura Y., Yoshimoto M., Murata Y., Shimoishi Y., Asai Y., Park E.Y., Sato K., Nakamura Y. (2007). Papaya seed represents a rich source of biologically active isothiocyanate. J. Agric. Food Chem..

[20] Hiraga Y., Ara T., Sato N., Akimoto N., Sugiyama K., Suzuki H., Kera K. (2021). Metabolic analysis of unripe papaya (*Carica papaya* L.) to promote its utilization as a functional food. Biosci. Biotechnol. Biochem..

[21] Lieb V.M., Esquivel P., Cubero Castillo E., Carle R., Steingass C.B. (2018). GC–MS profiling, descriptive sensory analysis, and consumer acceptance of Costa Rican papaya (*Carica papaya* L.) fruit purees. Food Chem..

[22] Govindachari T.R., Nagarajan K., Viswanathan N. (1965). Carpaine and pseudocarpaine. Tetrahedron Lett..

[23] Tang C.-S. (1979). New macrocyclic, Δ^1^-piperideine alkaloids from papaya leaves: Dehydrocarpaine I and II. Phytochemistry.

[24] Burdick E.M. (1971). Carpaine: An alkaloid of *Carica papaya*–its chemistry and pharmacology. Econ. Bot..

[25] Zunjar V., Dash R.P., Jivrajani M., Trivedi B., Nivsarkar M. (2016). Antithrombocytopenic activity of carpaine and alkaloidal extract of *Carica papaya* L. leaves in busulfan induced thrombocytopenic Wistar rats. J. Ethnopharmacol..

[26] Yaohai Z., Nian Z., Chengqiu W., Jing L.I., Xijuan Z., Bining J. (2023). Targeted screening and quantification of bioactive components of pomelo fruits at different maturity stages based on ultra–high performance liquid chromatography–quadrupole–tandem mass spectrometry. Food Ferment. Ind..

[27] Fuping L.I.U., Huihong L., Xi W., Dongkui C., Chizhang W.E.I., Hongming H., Nina W. (2024). Differential analysis of flesh compounds of grapefruit of guipuyou No. 1 at different maturation stages based on widely targeted metabolomics. Chin. J. Trop. Crops.

[28] Zhang B., Horvath S. (2005). A general framework for weighted gene co-expression network analysis. Stat. Appl. Genet. Mol. Biol..

[29] Langfelder P., Horvath S. (2008). WGCNA: An R package for weighted correlation network analysis. BMC Bioinform..

[30] Abe M., Katsumata H., Komeda Y., Takahashi T. (2003). Regulation of shoot epidermal cell differentiation by a pair of homeodomain proteins in *Arabidopsis*. Development.

[31] Peng Y., Liu Y., Wang Y., Geng Z., Qin Y., Ma S. (2024). Stomatal maturomics: Hunting genes regulating guard cell maturation and function formation from single-cell transcriptomes. J. Genet. Genom..

[32] Ohashi-Ito K., Bergmann D.C. (2006). *Arabidopsis* FAMA controls the final proliferation/differentiation switch during stomatal development. Plant Cell.

[33] Lee S.B., Suh M.C. (2018). Disruption of glycosylphosphatidylinositol-anchored lipid transfer protein 15 affects seed coat permeability in *Arabidopsis*. Plant J..

[34] Zhou J., Zhong R., Ye Z.-H. (2014). *Arabidopsis* NAC Domain Proteins, VND1 to VND5, Are Transcriptional Regulators of Secondary Wall Biosynthesis in Vessels. PLoS ONE.

[35] Taylor N.G., Scheible W.-R., Cutler S., Somerville C.R., Turner S.R. (1999). The irregular xylem3 Locus of *Arabidopsis* Encodes a Cellulose Synthase Required for Secondary Cell Wall Synthesis. Plant Cell.

[36] Wallner E.-S., López-Salmerón V., Belevich I., Poschet G., Jung I., Grünwald K., Sevilem I., Jokitalo E., Hell R., Helariutta Y. (2017). Strigolactone- and Karrikin-Independent SMXL Proteins Are Central Regulators of Phloem Formation. Curr. Biol..

[37] Wang B., Luo C., Li X., Jimenez A., Cai J., Chen J., Yu F. (2025). The FERONIA-RESPONSIVE TO DESICCATION 26 module regulates vascular immunity to *Ralstonia solanacearum*. Plant Cell.

[38] Cai J., Panda S., Kazachkova Y., Amzallag E., Li Z., Meir S., Aharoni A. (2024). A NAC triad modulates plant immunity by negatively regulating *N*-hydroxy pipecolic acid biosynthesis. Nat. Commun..

[39] Wang J., Li Y., Yang Y., Xiao C., Ruan Q., Li P., Kai G. (2023). Comprehensive analysis of OpHD-ZIP transcription factors related to the regulation of camptothecin biosynthesis in *Ophiorrhiza pumila*. Int. J. Biol. Macromol..

[40] Li R.C. (2023). Single-Cell Transcriptomics Reveals the Spatial Distribution of Ginsenoside Biosynthetic Pathways in the Shoot Tips and Leaves of *Gynostemma pentaphyllum*. Master’s Thesis.

[41] Li Y. (2021). Single-Cell Transcriptome Sequencing Reveals the Spatial Specificity of Monoterpenoid Indole Alkaloid Biosynthetic Pathways in *Catharanthus roseus* Leaves. Master’s Thesis.

[42] Cock P.J.A., Fields C.J., Goto N., Heuer M.L., Rice P.M. (2010). The Sanger FASTQ file format for sequences with quality scores, and the Solexa/Illumina FASTQ variants. Nucleic Acids Res..

[43] Chen S., Zhou Y., Chen Y., Gu J. (2018). fastp: An ultra-fast all-in-one FASTQ preprocessor. Bioinformatics.

[44] Bolger A.M., Lohse M., Usadel B. (2014). Trimmomatic: A flexible trimmer for Illumina sequence data. Bioinformatics.

[45] Kim D., Paggi J.M., Park C., Bennett C., Salzberg S.L. (2019). Graph-based genome alignment and genotyping with HISAT2 and HISAT-genotype. Nat. Biotechnol..

[46] Anders S., Huber W. (2010). Differential expression analysis for sequence count data. Genome Biol..

[47] Cantalapiedra C.P., Hernández-Plaza A., Letunic I., Bork P., Huerta-Cepas J. (2021). eggNOG-mapper v2: Functional annotation, orthology assignments, and domain prediction at the metagenomic scale. Mol. Biol. Evol..

[48] Mu H., Chen J., Huang W., Huang G., Deng M., Hong S., Ai P., Gao C., Zhou H. (2024). OmicShare tools: A zero-code interactive online platform for biological data analysis and visualization. iMeta.

[49] Shannon P., Markiel A., Ozier O., Baliga N.S., Wang J.T., Ramage D., Amin N., Schwikowski B., Ideker T. (2003). Cytoscape: A software environment for integrated models of biomolecular interaction networks. Genome Res..

[50] Livak K.J., Schmittgen T.D. (2001). Analysis of relative gene expression data using real-time quantitative PCR and the 2^−ΔΔCT^ method. Methods.

[51] Conde D., Triozzi P.M., Balmant K.M., Doty A.L., Miranda M., Boullosa A., Schmidt H.W., Pereira W.J., Dervinis C., Kirst M. (2021). A robust method of nuclei isolation for single-cell RNA sequencing of solid tissues from the plant genus *Populus*. PLoS ONE.

[52] Sunaga-Franze D.Y., Muino J.M., Braeuning C., Xu X., Zong M., Smaczniak C., Yan W., Fischer C., Vidal R., Kliem M. (2021). Single-nucleus RNA sequencing of plant tissues using a nanowell-based system. Plant J..

[53] Qiu X., Mao Q., Tang Y., Wang L., Chawla R., Pliner H.A., Trapnell C. (2017). Reversed graph embedding resolves complex single-cell trajectories. Nat. Methods.

[54] Chen T., Chen X., Zhang S., Zhu J., Tang B., Wang A., Dong L., Zhang Z., Yu C., Sun Y. (2021). The Genome Sequence Archive family: Toward explosive data growth and diverse data types. Genom. Proteom. Bioinform..

[55] CNCB-NGDC Members and Partners (2025). Database resources of the National Genomics Data Center, China National Center for Bioinformation in 2025. Nucleic Acids Res..

