# Supplementary material for: Deciphering Alkaloid Bitter Compounds and Relevant Transcription Factors in Papaya
Source: Int J Mol Sci. 2026 Apr 11;27(8):3438. doi: 10.3390/ijms27083438 (PMC13116859; doi:10.3390/ijms27083438)
Supplement: Supplementary file 1 [file ijms-27-03438-s001.zip › ijms-4192793-supplementary/Supplementary Figures and Tables/Supplementary Figure S3.pdf]

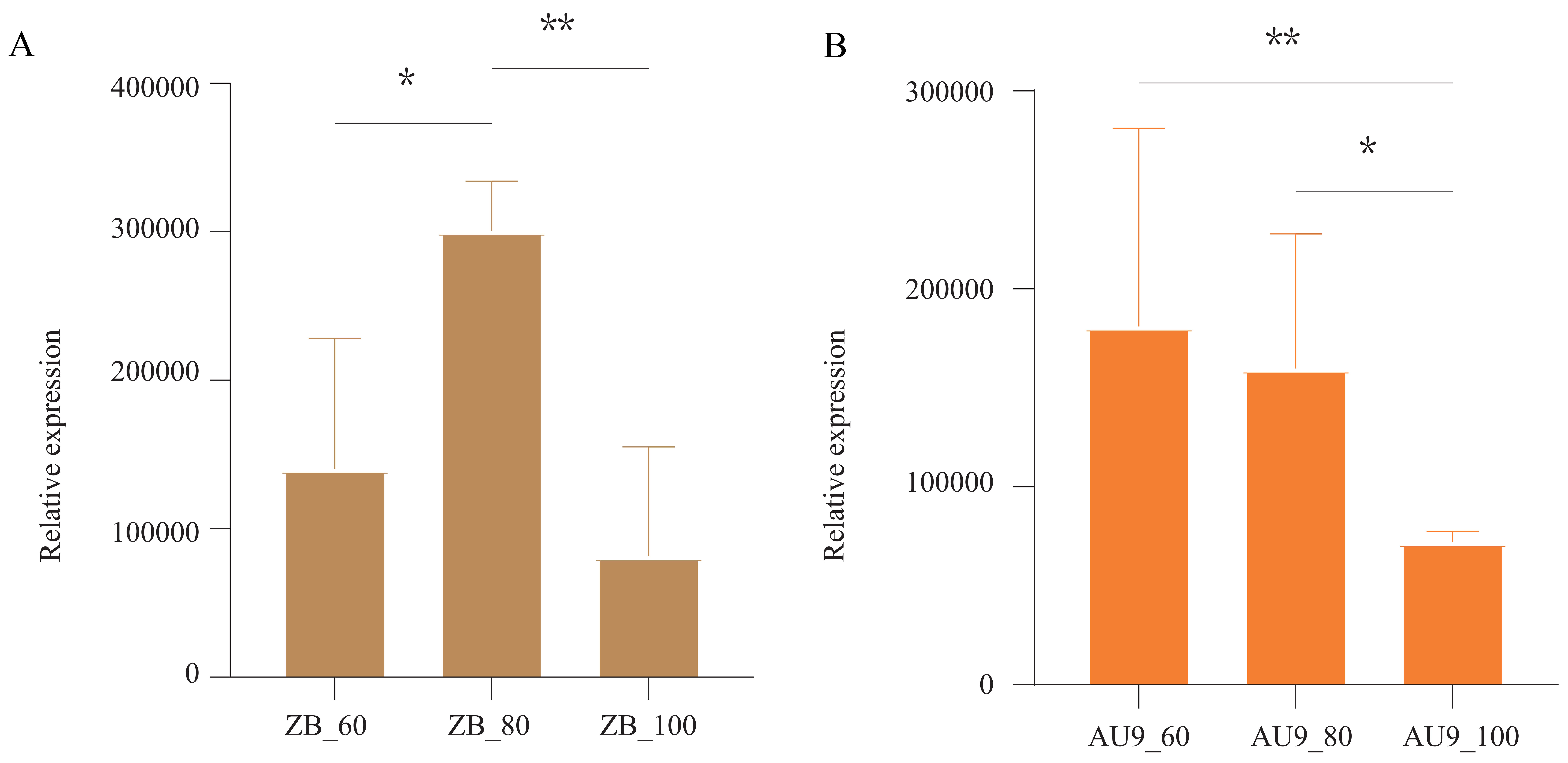

Figure S3. Dynamic changes in total alkaloid content during different ripening stages in ‘Zhongbai’ (A) and AU9 (B). Error bars indicate the standard error of three biological replicates. Significance levels: * $p < 0.05$, ** $p < 0.01$.
