# Supplementary material for: Deciphering Alkaloid Bitter Compounds and Relevant Transcription Factors in Papaya
Source: Int J Mol Sci. 2026 Apr 11;27(8):3438. doi: 10.3390/ijms27083438 (PMC13116859; doi:10.3390/ijms27083438)
Supplement: Supplementary file 1 [file ijms-27-03438-s001.zip › ijms-4192793-supplementary/Supplementary Figures and Tables/Supplementary Figure S5.pdf]

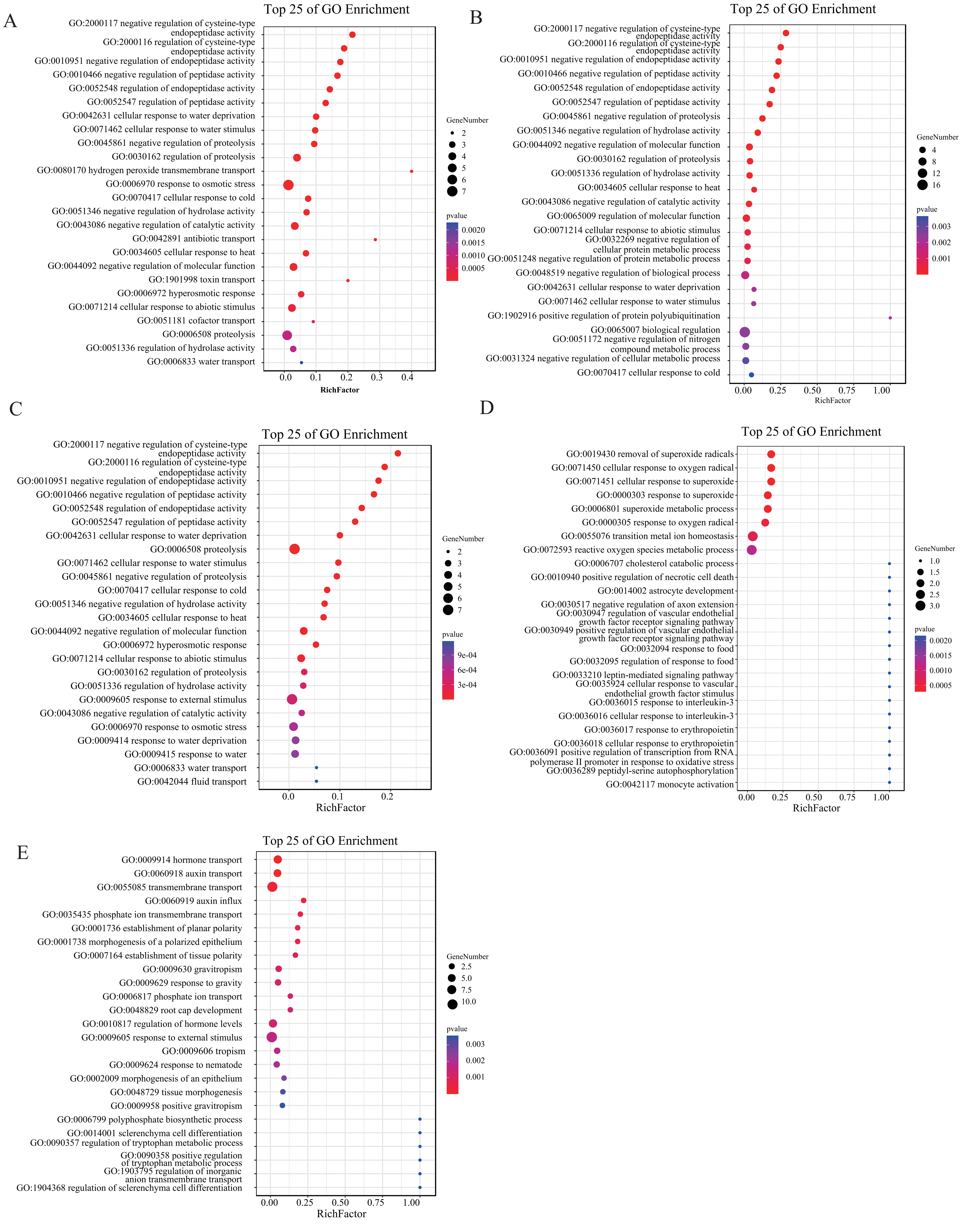

Figure S5. GO enrichment analysis of differentially expressed genes in cell clusters. (A) GO annotation of stem cell cluster 3. (B) GO annotation of stem cell cluster 4. (C) GO annotation of fibrous strands cell cluster 1. (D) GO annotation of fibrous strands cell cluster 6. (E) GO annotation of fibrous strands cell cluster 5.
