# Supplementary material for: Deciphering Alkaloid Bitter Compounds and Relevant Transcription Factors in Papaya
Source: Int J Mol Sci. 2026 Apr 11;27(8):3438. doi: 10.3390/ijms27083438 (PMC13116859; doi:10.3390/ijms27083438)
Supplement: Supplementary file 1 [file ijms-27-03438-s001.zip › ijms-4192793-supplementary/Supplementary Figures and Tables/Supplementary Figure S6.pdf]

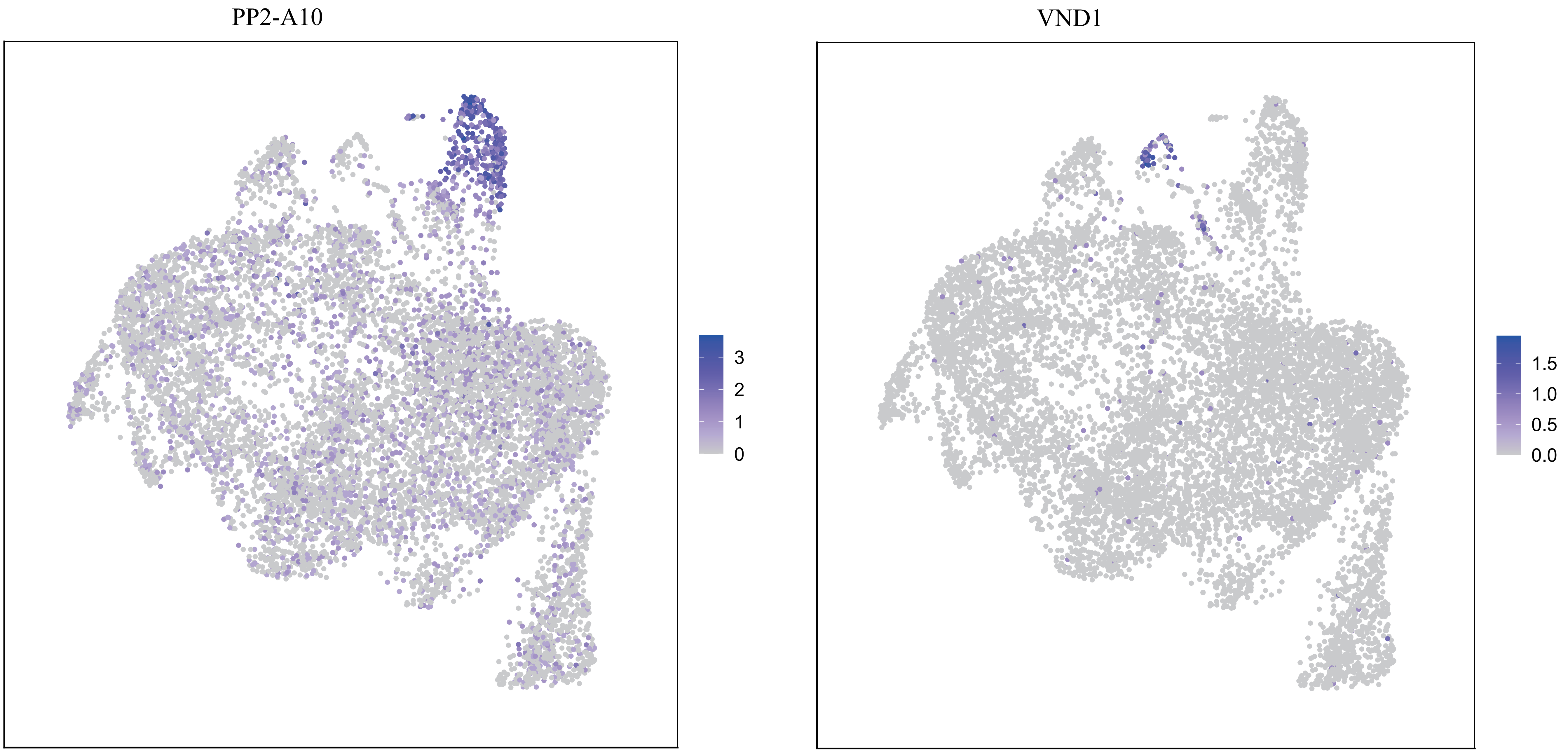


Figure S6. UMAP visualization of marker genes in papaya fibrous strands. Each dot denotes a nucleus.
