# Supplementary material for: Deciphering Alkaloid Bitter Compounds and Relevant Transcription Factors in Papaya
Source: Int J Mol Sci. 2026 Apr 11;27(8):3438. doi: 10.3390/ijms27083438 (PMC13116859; doi:10.3390/ijms27083438)
Supplement: Supplementary file 1 [file ijms-27-03438-s001.zip › ijms-4192793-supplementary/Supplementary Figures and Tables/Supplementary Figure S7.pdf]

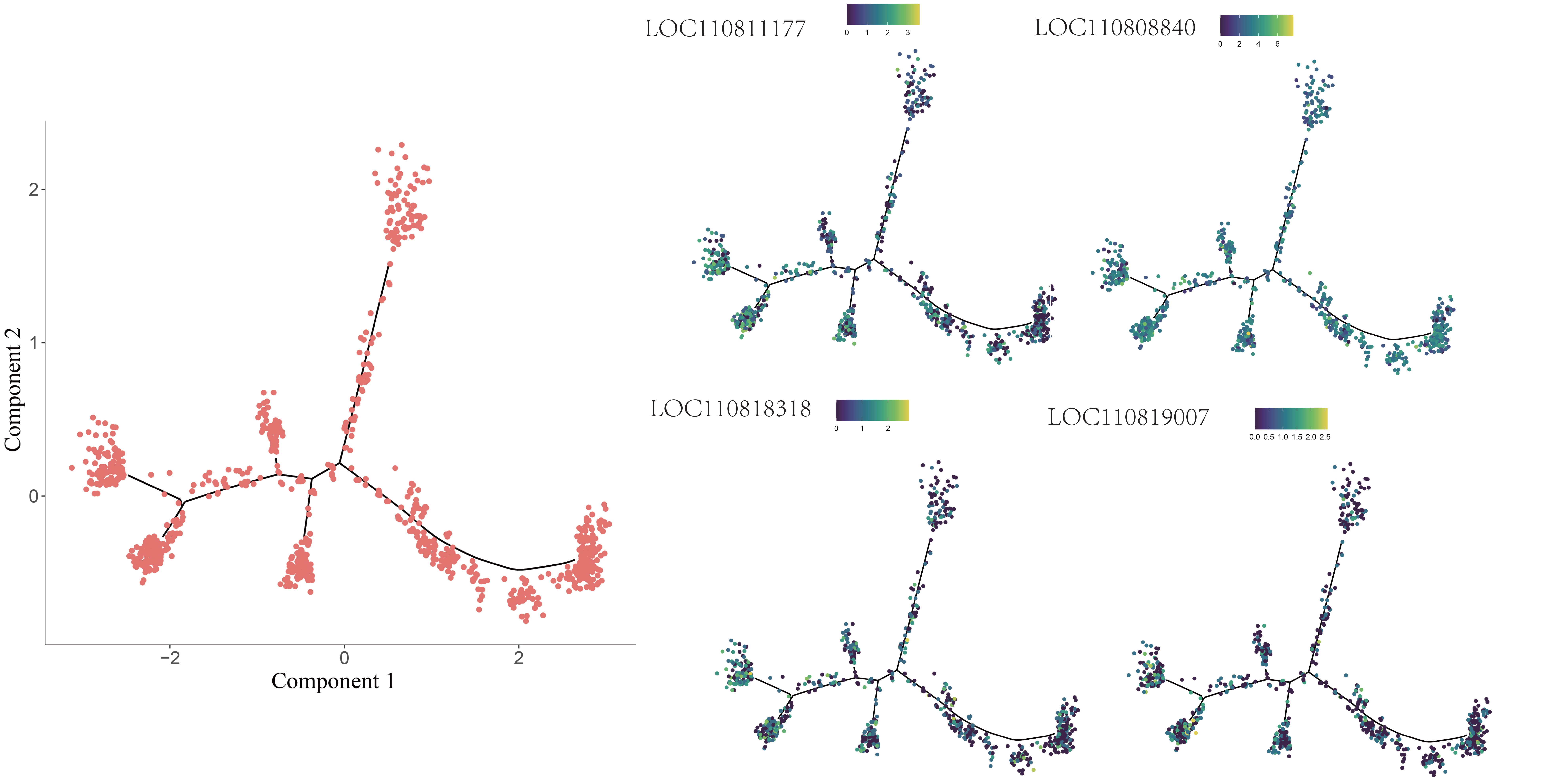

Figure S7. Differentiation trajectory of epidermal cells in papaya stem. A: Epidermal cell trajectory visualization. Each dot represents one nucleus. B: Trajectory visualization of epidermal cell–specific genes within the lightcyan module. Each dot denotes a nucleus.
