# Supplementary material for: Deciphering Alkaloid Bitter Compounds and Relevant Transcription Factors in Papaya
Source: Int J Mol Sci. 2026 Apr 11;27(8):3438. doi: 10.3390/ijms27083438 (PMC13116859; doi:10.3390/ijms27083438)
Supplement: Supplementary file 1 [file ijms-27-03438-s001.zip › ijms-4192793-supplementary/Supplementary Figures and Tables/Supplementary Figure S8.pdf]

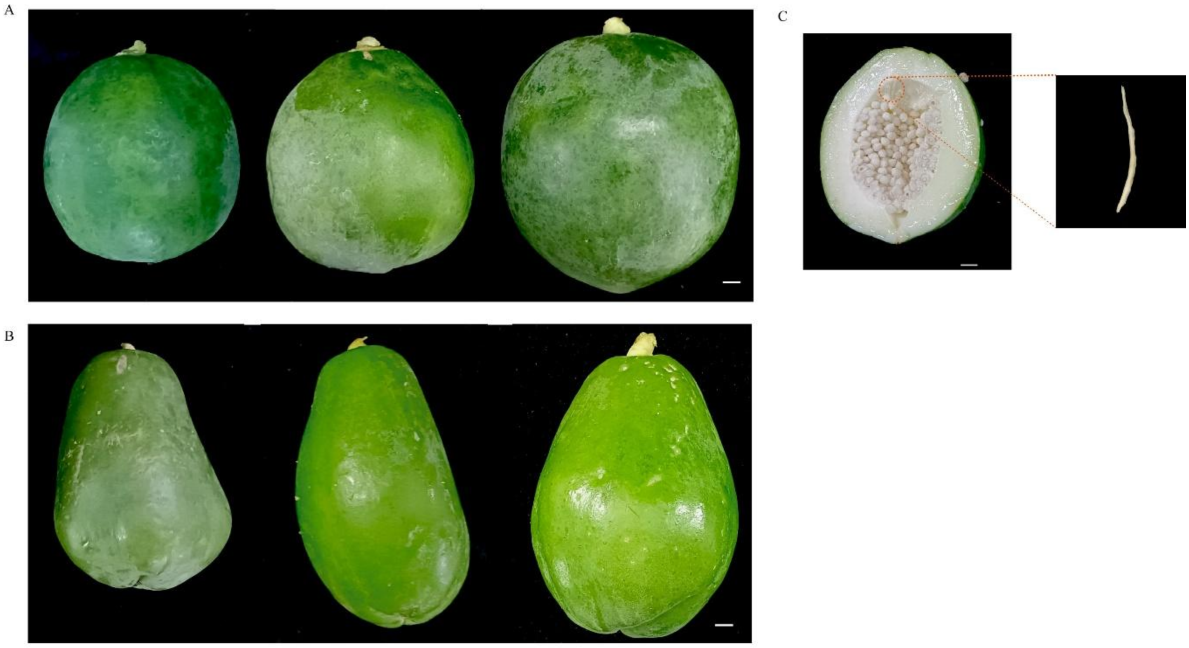


Figure S8. Papaya fruit at different ripening stages. (A-B): AU9 (upper panel) and 'Zhongbai' (lower panel) at 60, 80, 100 days (left to right) after pollination. The scale bar at the bottom right corner preoresents 1 cm.
