# Supplementary figures and images for: Deciphering Alkaloid Bitter Compounds and Relevant Transcription Factors in Papaya
Source: Int J Mol Sci. 2026 Apr 11;27(8):3438. doi: 10.3390/ijms27083438 (PMC13116859; doi:10.3390/ijms27083438)

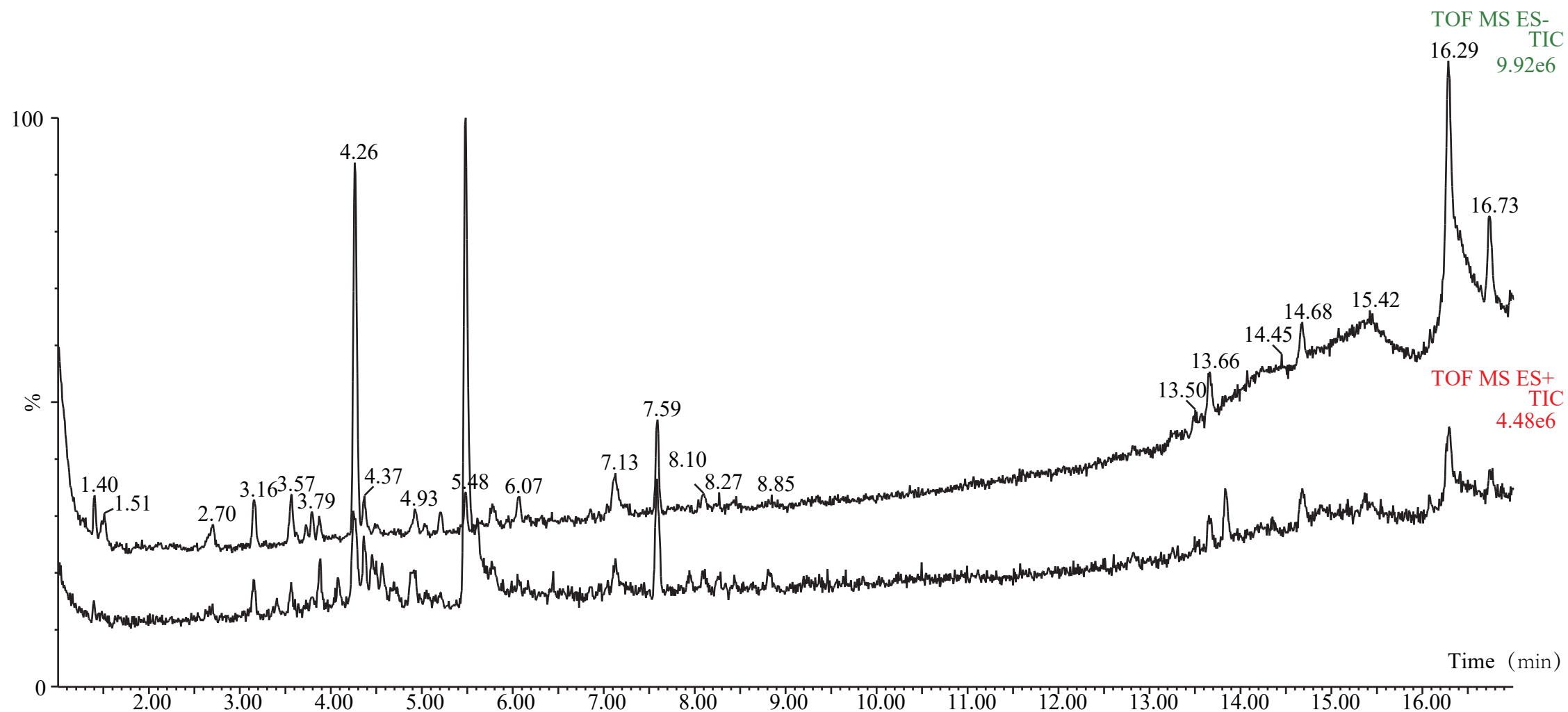

Figure S1. Total ion chromatograms of papaya fibrous strands in positive and negative ion modes.

Supplement: Supplementary file 1 [file ijms-27-03438-s001.zip › ijms-4192793-supplementary/Supplementary Figures and Tables/Supplementary Figure S1.pdf]

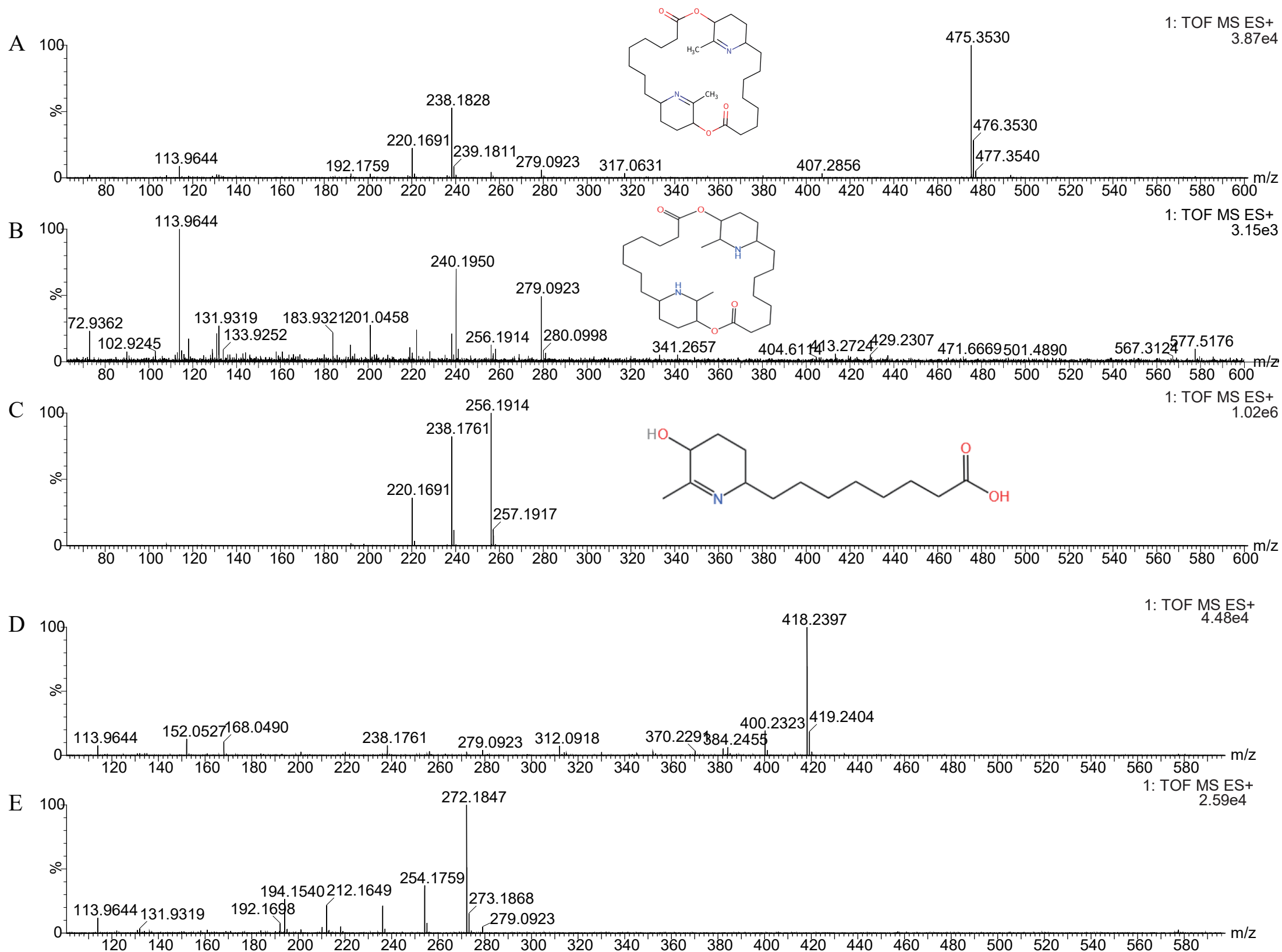

Figure S2. The MS/MS spectrum of papaya alkaloids.

Supplement: Supplementary file 1 [file ijms-27-03438-s001.zip › ijms-4192793-supplementary/Supplementary Figures and Tables/Supplementary Figure S2.pdf]

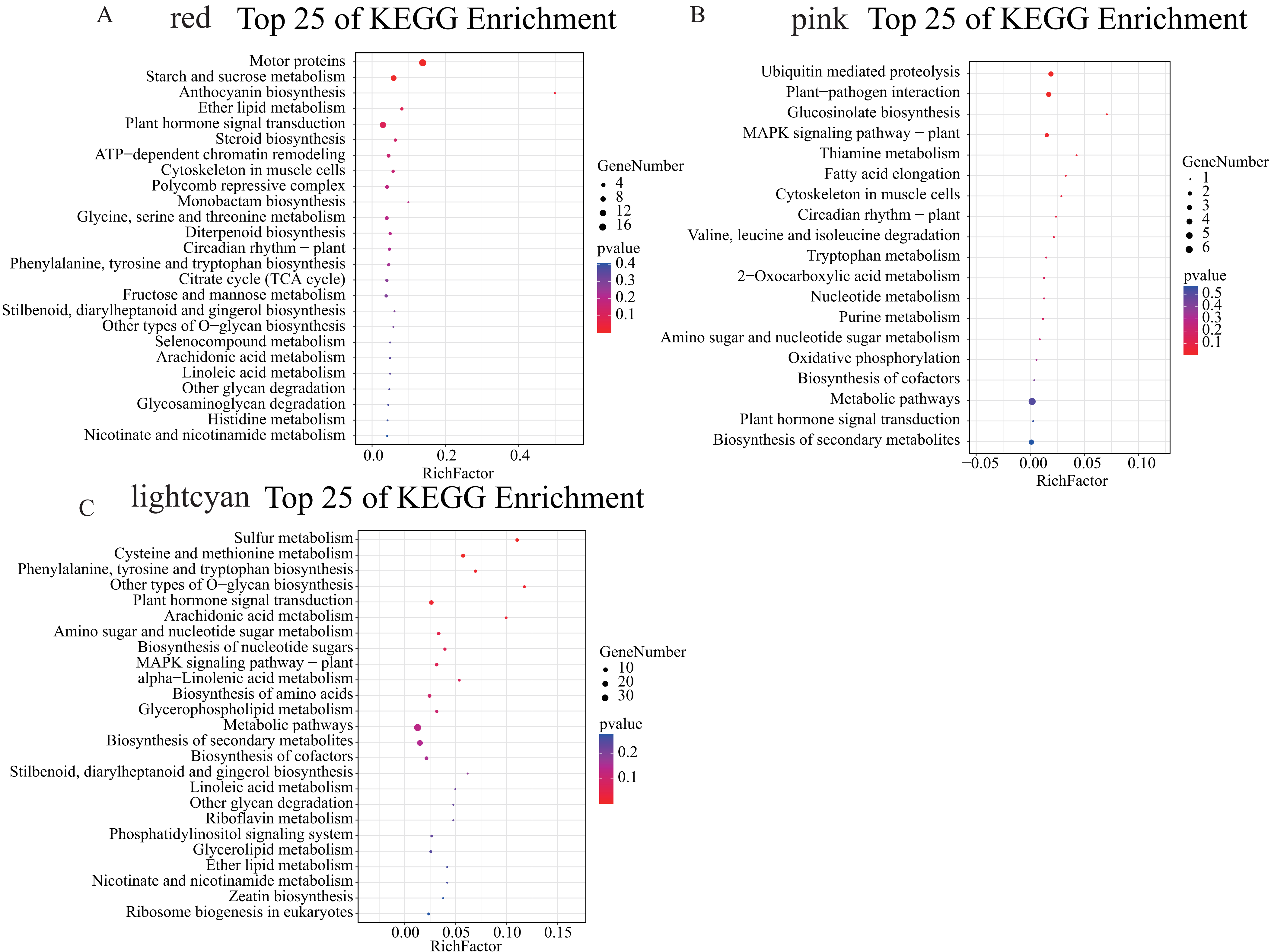

Figure S4. red (A), pink (B), and lightcyan (C) KEGG enrichment analysis

Supplement: Supplementary file 1 [file ijms-27-03438-s001.zip › ijms-4192793-supplementary/Supplementary Figures and Tables/Supplementary Figure S4.pdf]
